# First Records of Morphological Diversity and Ecology of Periphytic Cyanobacteria from Tukun River, Penang Forest Reserve, Malaysia

**DOI:** 10.21315/tlsr2020.31.1.6

**Published:** 2020-04-07

**Authors:** Asmimie Asmawarnie Azizan, Ranina Radzi, Wan Maznah Wan Omar, Peter Convey, Faradina Merican Mohd Sidik Merican

**Affiliations:** 1 School of Biological Sciences, Universiti Sains Malaysia, 11800 USM Pulau Pinang, Malaysia; 2 British Antarctic Survey, Natural Environment Research Council (NERC), High Cross, Madingley Road, Cambridge CB3 0ET, United Kingdom

**Keywords:** Cyanobacteria, Malaysia, Periphytic, Stream, National Park, Sianobakteria, Malaysia, Perifitik, Sungai, Taman Negara

## Abstract

Despite the abundance of streams and rivers in Malaysia, the algal communities of these lotic ecosystems have remained largely unstudied. In a one-year floristic survey conducted from December 2014, 24 cyanobacterial morphospecies were identified for the first time from Tukun River, Penang Forest Reserve. Ten morphospecies were identified directly from field specimens while the remaining 14 morphospecies were identified only in cultures derived from the field samples. A total of 17 morphospecies; *Leptolyngbya* cf. *boryana, L.* cf. *foveolarum, L. valderiana, Chroococcus* cf. *cohaerens, C.* cf. *disperses, C.* cf. *membraninus, C.* cf. *minutus, C.* cf. *varius, Gloeocapsopsis* cf. *crepidinum, Geitlerinema* cf. *tenuius, Phormidium simplicissimum, Dolichospermum* sp., *Fischerella* sp., *Homoeoptyche repens, Nematoplaca inscrustans, Scytonema hofmanii* and *S. stuposum* are new records for Malaysia. Crusts were the most dominant macroscopic forms (seven morphospecies) followed by mats (three morphospecies). *Scytonema* was the most frequently encountered genus, occurring at 8/9 sampling sites. The presence of heterocytous cyanobacteria (*S. stuposum*, *S. hofmanni*) in 8/9 sampling sites is consistent with the low nitrate levels (< 0.74 mg/L) recorded throughout the study stream. Chroococcales were dominant in both upper and middle parts of the stream. The morphospecies present showed distinct distribution patterns despite apparently minimal variations in ecological parameters such as temperature, dissolved oxygen, pH and conductivity between the sampling sites. This study provides important new baseline information in understanding the diversity of periphytic cyanobacteria not only in Penang Island but more widely in Malaysia. This information can make a useful contribution in biomonitoring stream health.

## Abstract (ms)

Meskipun terdapat banyak jeram dan sungai di Malaysia, komuniti alga ekosistem lotik ini masih belum banyak dipelajari. Dalam satu penyelidikan floristik selama setahun yang dijalankan semenjak Disember 2014, 24 morfospesies sianobakteria telah dikenal pasti buat pertama kalinya dari Sungai Tukun, Hutan Simpan Pulau Pinang. Sepuluh morfospesies telah dikenal pasti secara langsung dari spesimen lapangan, sementara 14 morfospesies selebihnya hanya dikenal pasti di dalam media kultur yang dikulturkan dengan spesimen dari lapangan. Sejumlah 17 morfospesies; *Leptolyngbya* cf. *boryana, L.* cf. *foveolarum, L. valderiana, Chroococcus* cf. *cohaerens, C.* cf. *disperses, C.* cf. *membraninus, C.* cf. *minutus, C.* cf. *varius, Gloeocapsopsis* cf. *crepidinum, Geitlerinema* cf. *tenuius, Phormidium simplicissimum, Dolichospermum* sp., *Fischerella* sp., *Homoeoptyche repens, Nematoplaca inscrustans, Scytonema hofmanii* dan *S. stuposum* adalah rekod untuk Malaysia. Kerak adalah bentuk makroskopik yang paling dominan (tujuh morfospesies) diikuti oleh hamparan (tiga morfospesies). *Scytonema* adalah genus yang paling kerap dijumpai di tapak persampelan 8/9. Kehadiran sianobakteria heterocytes (*S. stuposum*, *S. hofmanni*) di tapak persampelan 8/9 adalah konsisten dengan tahap nitrat rendah (< 0.74 mg/L) yang dicatatkan sepanjang kajian. Chroococcales dominan di bahagian atas dan tengah sungai. Morfospesies yang hadir menunjukkan corak taburan yang berbeza meskipun terdapat variasi yang sangat kecil dalam parameter ekologi seperti suhu, oksigen terlarut, pH dan kekonduksian antara tapak sampel. Kajian ini memberikan maklumat penting mengenai kepelbagaian sianobakteria perifitik bukan sahaja di Pulau Pinang tetapi juga di Malaysia. Maklumat daripada penyelidikan ini dapat digunakan untuk biopemantauan kesihatan sungai kita.

**Highlights**

- 24 morphospecies of periphytic cyanobacteria have been recorded for the first time in Tukun River, Penang Forest Reserve.
- More than 70% of the morphospecies recorded are new records for Malaysia.
- Knowledge on the diversity and distribution of periphytic cyanobacteria can be useful in monitoring stream health.

## INTRODUCTION

Cyanobacteria are amongst the most important periphytic algae community members in streams and rivers globally (Perona *et al.* 1998; Lindstrøm *et al.* 2004; Sherwood 2006; Yang *et al.* 2009; Krupek & Branco 2012; Loza *et al.* 2013). They can be useful as biomonitoring indicators in lotic habitats (Loza *et al.* 2013), providing valuable indicators of the overall health of these ecosystems, and they are particularly useful as assessment tools due to their rapid responses to environmental stress (Omar 2010). They have vital roles in stream nitrogen, carbon, and oxygen dynamics (Vincent 2000), and are the main primary producers in aquatic food webs (Scott & Marcarelli 2012), also contributing to substrate stabilisation (Casamatta & Hašler 2016).

Notwithstanding their importance, information on biodiversity of tropical cyanobacteria is lacking for many ecosystems and habitats. The present knowledge of their diversity in the tropics is estimated to be less than 10% of those probably present (Komárek 2006) with perhaps as little as 3% addressed in published material on the subject (Dvořák *et al*. 2015). This is particularly true for the lotic ecosystems in Malaysia.

The first study on algae in Malaysia was conducted by Patrick (1948) and focused on freshwater diatoms from streams in North Malaysia. Subsequent studies included work on desmids (Prowse 1957), Euglenophytes (Prowse 1958), Chrysophytes (Prowse 1960), Rhodophytes (Kumano 1978), Bacillariophytes and Chlorophytes (Anton 1990; Maznah *et al.* 2000; Merican *et al.* 2006). Cyanobacteria have only been recorded as the least common community component co-occurring with diatoms and green algae. Existing studies have generally only identified prominent cyanobacterial taxa to the genus level, with very limited taxonomic information provided (Anton 1990; Maznah *et al.* 2000; Merican *et al.* 2006). In comparison, numerous studies have been conducted on lentic planktonic cyanobacteria communities in Malaysia (Harith & Hasan 2007, 2009, 2011; Majit *et al.* 2010; Florence 2011; Sinang *et al.* 2015). Both periphytic and planktonic algae communities have important roles as primary producers in aquatic ecosystems.

Environmental factors influencing the distribution of cyanobacteria in Malaysian streams are unknown, although cyanobacteria generally are known to have ability to detect and respond to various environmental drivers. According to Dolman *et al.* (2012), in low nitrate content waters the capacity of some cyanobacterial species to fix nitrogen allows them to outcompete others that lack this ability. Globally, their success in colonising a wide range of habitats is assisted by their resistance to extremes of temperature and desiccation and their modest nutrient requirements. Most nitrogen-fixing cyanobacteria can thrive photoautotrophically in the absence of combined nitrogen (Carr & Whitton 1982). Cyanobacteria are also able to grow under low-light conditions due to their accessory pigments, known as phycobilins, in particular phycocyanin and phycoerythrin (Loeb & Reuter 1981; Reuter & Axler 1992); this allows them to colonise shaded waters.

In Malaysia, cyanobacteria remain one of the most understudied freshwater microbial groups. Furthermore, increased knowledge of their distribution in relation to environmental parameters will enhance the use of cyanobacterial assemblages for biomonitoring of Malaysian stream health. Here we present the first extensive study of cyanobacterial diversity for a Malaysian river ecosystem.

## MATERIALS AND METHODS

### Site Description

**Figure 1:**
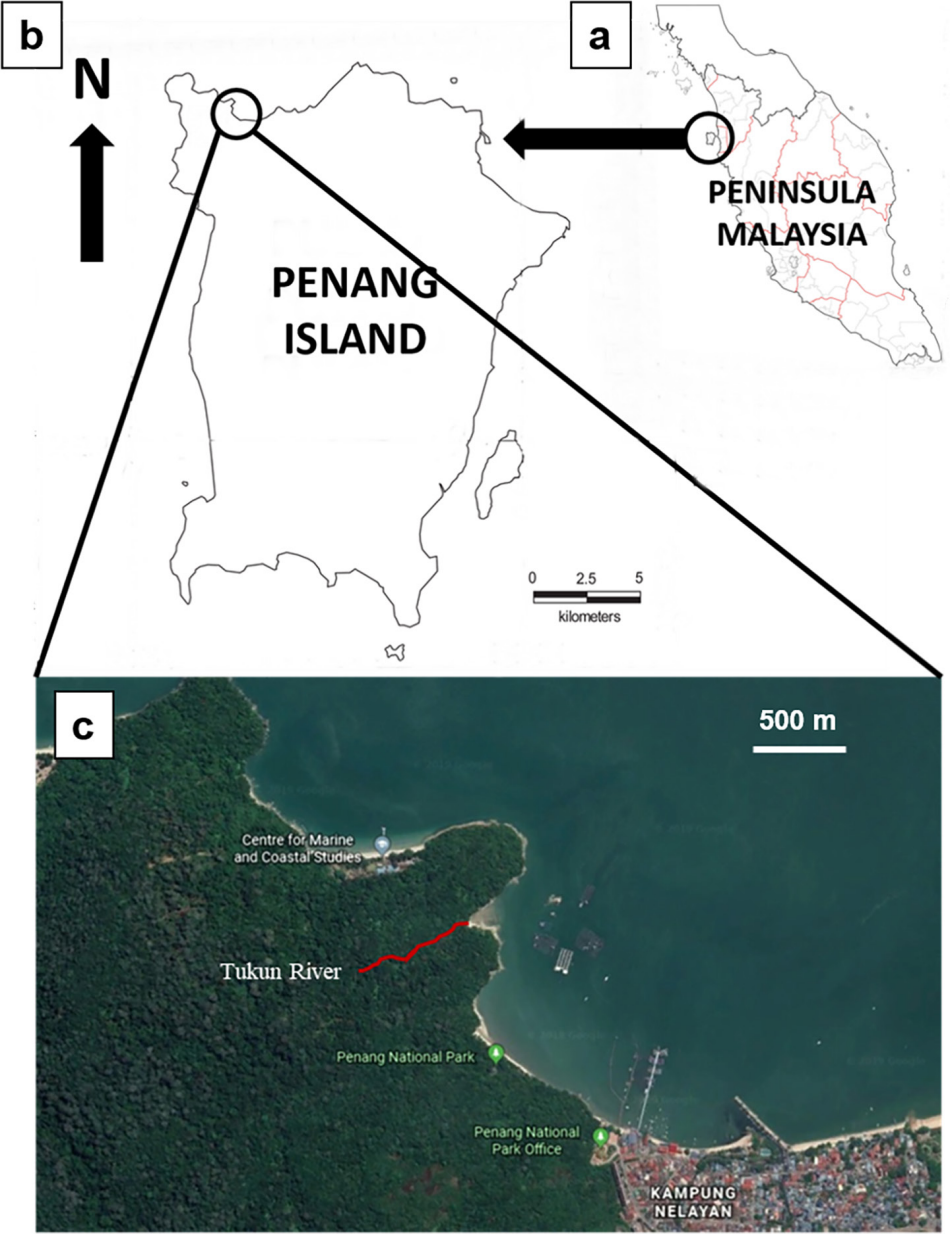
Locality map of study site: (a) Peninsula Malaysia and location of Penang Island off the north-western coast circled in black; (b) location of Penang National Park circled in black; (c) the study site shown in red. Scale bars: 500 m for (a); 4 km for (b) (Modified from Google Maps).

The study area is in the Penang National Park in the north-west of Penang Island (5° 28′ N; 100° 12′ E) (Fig. 1). The area was formerly known as Pantai Acheh Forest Reserve. It primarily consists of an intact and generally undisturbed lowland dipterocarp rainforest. Penang National Park was gazetted under the National Parks Act 1980, No. 226 and has a total area of 2,563.0 ha, of which 1,182.6 ha are terrestrial and 1,379.3 ha are marine. Penang National Park is the smallest national park in Malaysia and one of the smallest national parks in the world (Ahmad 2007). The Park is also used for public recreational activities. Construction of facilities available for visitors has been focused on two areas, the lower part of Tukun River and Pantai Kerachut, before the area was gazetted as a national park in 2003 (Ahmad 2007). Average annual rainfall in the park is 2,600 mm. Rainfall pattern is strongly influenced by the north-east Monsoon, and a dry season usually occurs from December to March. Temperature ranges between 23°C–30°C (Ahmad 2007).

**Figure 2:**
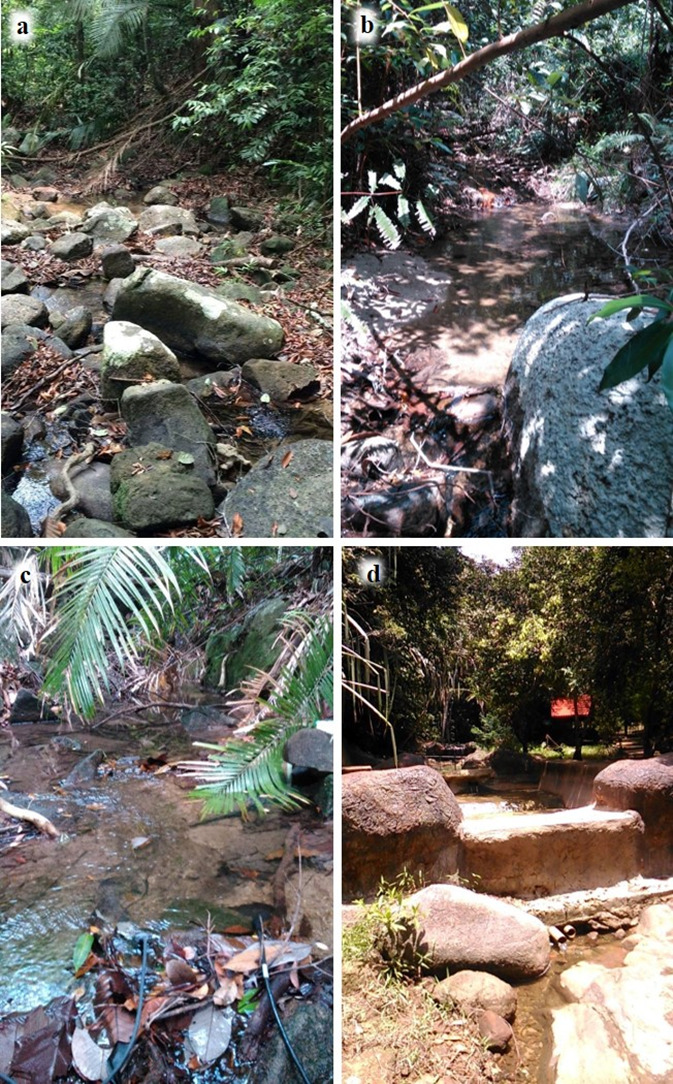
(a) Upper stream; stream bed substratum mainly consisting of boulders and cobbles; (b) Middle stream; stream bed substratum consisting of mainly pebbles and gravel with occasional boulders and cobbles; (c) Middle stream; some localities with sand and silt as the dominant substratum; (d) Lower stream; presence of artificial substrata due to construction of public picnic area.

Tukun River is a small stream, approximately 700 m long. Sampling locations consisted of three sites, located in the upper stream, middle stream and lower stream. Sampling locations were numbered from 1–9 with 1 being at the upstream end of the site in Tukun River and 9 at the downstream end. The geological substrata of the stream included boulders, cobbles, bedrock, sand and silts (Fig. 2). Stream substrata were classified based on a modified Wentworth scale: bedrock > 4000 mm, boulders > 256–4000 mm, cobbles > 64–256 mm, pebbles > 16–64 mm, gravel > 2–16 mm, sand > 0.063–2 mm, silt < 0.063 mm (Harding *et al.* 2009). The degree of shading due to riparian vegetation was characterised following the categories of Harding *et al.* (2009): unshaded, 0%–30% shade; partly shaded, 30%–60% shade; shaded, > 60% shade. Description of water flow within each sampling site (runs, riffles and pools) was again based on the definitions of Harding *et al.* (2009).

### Sample Collection

A floristic survey was carried out between December 2014 and November 2015. All accessible locations along the stream were included in the study. A total of nine sampling sites were identified for monthly survey. Samples of visible growth of cyanobacteria were collected from different substrata, including bedrock, boulders, cobbles, pebbles, gravels, sand and silt. Crusts were scraped from rock surfaces using clean spatulas. Mats, gelatinous colonies, and other macroalgae and macrophytes with possible epiphytic cyanobacteria were also collected. Each sample was immediately stored in a separate sterile polycarbonate screw-top container (60 mL) with water from the collecting site and transported back to the laboratory for further analysis.

### Cultivation and Identification

The specimens were cultivated in BG-11 and BG-11∘ (lacking chemically combined nitrogen) 1% agarised media (Rippka *et al.* 1979) supplemented with 100 μg/mL cycloheximide to prevent eukaryotic growth in cultures (Bolch & Blackburn 1996). Plate cultures of mat samples were examined after one week of incubation (25°C; 12:12 h light:dark from fluorescent lamp; ±1450 lx). Field specimens and cultured strains were observed using an Olympus BX50 microscope (Olympus America Inc., Center Valley, PA) at 100x–2000x magnification. Morphological features, including vegetative cell width and length, heterocyte and akinete width, length and shape, and filament or trichome width were measured. Size measurements were made for 30 replicates randomly chosen specimens for each morphospecies. Identification followed the taxonomic system of Komárek and Anagnostidis (1999, 2002) and Komárek (2013). Identifications were carried out to the lowest taxonomic level possible. Where uncertainty existed, the abbreviation “cf.” (Latin *conferatur*: to compare with) was used.

### Water Quality Assessment

Physicochemical parameters were measured *in situ* at each location during each sampling occasion. Temperature, dissolved oxygen, total suspended solids, salinity, nitrate and ammonium concentration were measured using YSI professional multi probes. pH was measured using a Hach 5465010 Sension 156 pH meter. For orthophosphate analysis, three replicates of 120 mL water from each site were collected into separate 120 mL acid-cleaned polyethylene bottles on each sampling occasion. These were then transported back to the laboratory on ice in a cool box. Orthophosphate was measured by using the ascorbic acid method or the Murphy-Riley method based on APHA standards (Eaton *et al.* 2005; Rice *et al.* 2012).

### Statistical Analysis

Canonical correspondence analysis (CCA) was carried out using CANOCO 5 software to analyse the relationships between environmental parameters and cyanobacteria community composition (R Core Team 2014; Ter Braak & Smilauer 2012). Variables analysed included nutrient contents (nitrate, ammonium and phosphate), temperature, dissolved oxygen, total suspended solids, pH, type of substratum (bedrock, boulder, cobble, gravel, pebble, sand and silt), shading (shaded, partly shaded and unshaded), and type of water flow (run, riffle and pool).

## RESULTS

### Morphospecies Identification and Assessment

Twenty-four morphospecies, including five Synechococcales, ten Chroococcales, two Oscillatoriales and seven Nostocales were identified from Tukun River, for which no previous records of cyanobacteria exist (Table 1). Seventeen of these; *Leptolyngbya* cf. *boryana, L.* cf. *foveolarum, L. valderiana, Chroococcus* cf. *cohaerens, C.* cf. *disperses, C.* cf. *membraninus, C.* cf. *minutus, C.* cf. *varius, Gloeocapsopsis* cf. *crepidinum, Geitlerinema* cf. *tenuius, Phormidium simplicissimum, Dolichospermum* sp., *Fischerella* sp., *Homoeoptyche repens, Nematoplaca inscrustans, Scytonema hofmanii* and *S. stuposum* are new records for Malaysia.

**Figure 3:**
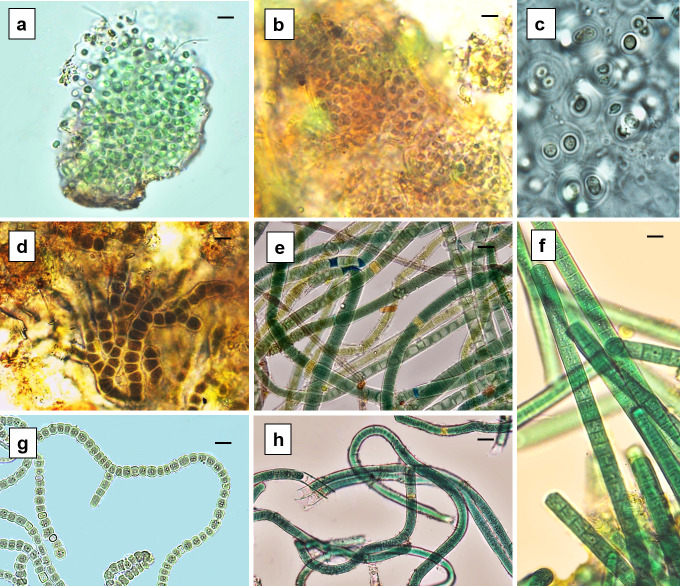
Examples of morphospecies identified; (a) *Aphanocapsa* cf. *musicola;* (b) *Microcystis* cf. *firma;* (c) *Gloeocapsa* cf. *rupicola;* (d) *Homoeoptyche repens;* (e) *Scytonema stuposum;* (f) *Phormidium simplicissimum;* (g) *Fischerella* sp.; (h) *Scytonema hofmanni.*

Crusts were the most dominant macroscopic growth form compared to mats and were the most diverse with seven taxa identified. Slimy black crust was the dominant crust type encountered compared to circular black crust and blue-green crust throughout the study. The morphospecies that dominated each crust type were different. Diversity present in the slimy black crust consisted of *Aphanocapsa* cf. *musicola* (Fig. 3)*, Microcystis* cf. *firma, Chroococcus* cf. *cohaerens, Nematoplaca inscrustans* and *Homoeoptyche repens* (Fig. 3), and this crust type was the most diverse with five morphospecies co-occuring within the same crust in site 2 and site 3 in the upper stream. The circular black crust and blue-green crust were each dominated by a single species, *Gloeocapsopsis* cf. *crepidinum* and *Scytonema hofmanni*, respectively. A total of three morphospecies was identified from the three other mat types collected in the sampling area: slimy blue-green mat (*Leptolyngbya valderiana*), slimy and coherent blue-green mat (*Phormidium simplicissimum*) (Fig. 3) and thin, woolly blue-green mat (*Scytonema stuposum*); these mat types occurred infrequently across the study area.

### Distribution Patterns of Cyanobacteria Across the Study Sites

A variety of cyanobacteria morphological types were identified across the study sites examined (Table 2). Some were recorded from a very limited range of sites, or single sites alone. *Gloeocapsopsis* cf. *crepidinum*, *Leptolyngbya valderina* and *Phormidium simplicissimum* were found only in sites 4, 9 and 5, respectively. *Geitlerinema* cf. *crepidinum* is epilithic, occurring in a partly shaded area in the middle stream. *Leptolyngbya. valderina* is epipelic and occurred in an unshaded area of the lower stream, and *P. simplicissimum*, which is also epipelic, occurred in a partly shaded area of the middle stream.

Other species occurred more widely. *Scytonema hofmanni* was recorded from all three sites in the upper stream and two of three sites in the middle stream, and *S. stuposum* was recorded in two sites from the middle stream and one site from the lower stream. These nitrogen-fixing cyanobacteria were the most widespread morphospecies encountered and were also widely distributed on various types of substrata and under different characteristics of water flow. Representatives of Chrooccocales occurred mostly in the upper and middle streams. These unicellular cyanobacteria were usually found in epilithic crusts on stable substrata such as boulders and cobbles.

### Environmental Parameters

**Figure 4:**
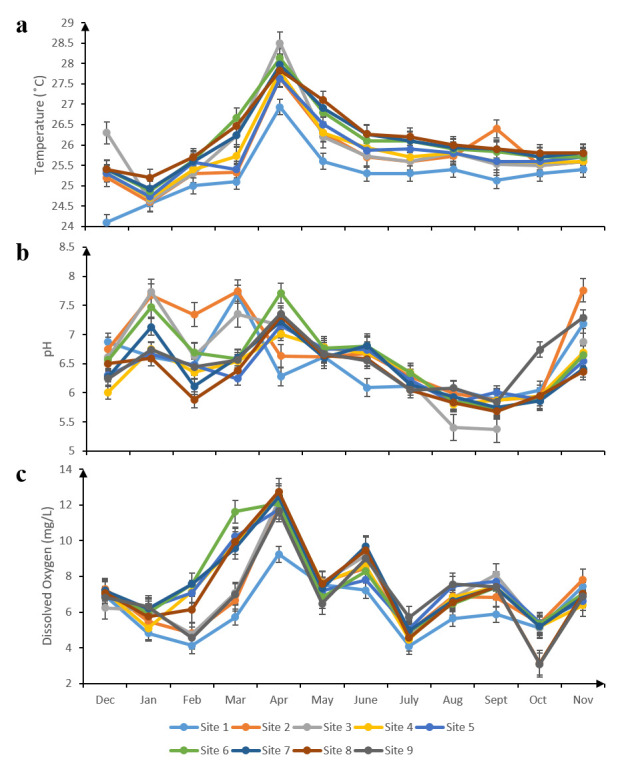
(a) Temperature; (b) pH; and (c) dissolved oxygen concentration in Tukun River at each sampling site between December 2014 and November 2015.

**Figure 5:**
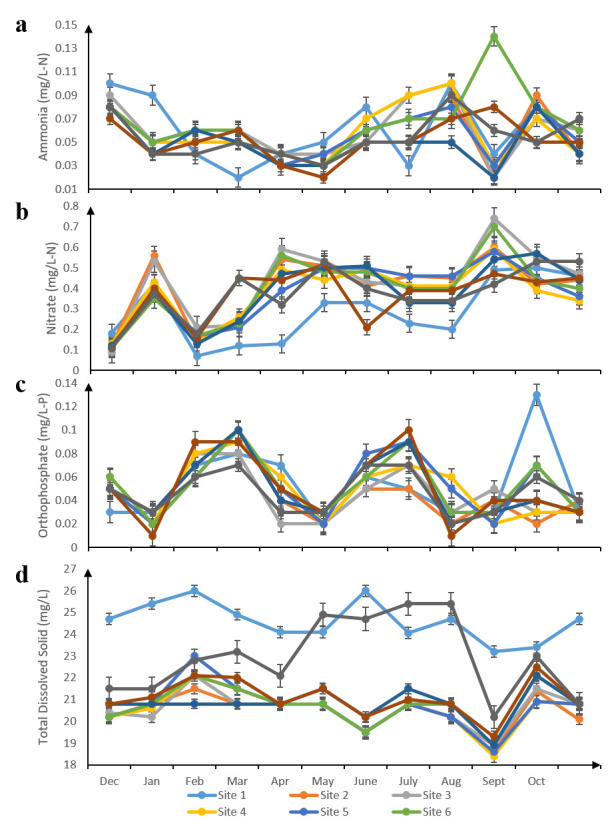
Nutrient concentrations at each sampling site between December 2014 and November 2015.

The highest water temperature (29.57°C) was recorded in April at site 9 while the lowest temperature (24.57°C) was recorded in January at site 1 (Fig. 4a). The highest pH value (7.76) was recorded in November at site 2 while the lowest value (5.68) was recorded in September at site 8 (Fig. 4b). Dissolved oxygen concentration was highest in April 2015 (12.53 mg/L) at site 4 and lowest (3.08 mg/L) in October at site 9 (Fig. 4c). Ammonium concentration was highest (0.1 mg/L) in December at site 1 and in August at sites 1, 3 and 4. The lowest value, 0.02 mg/L, was recorded at site 1 in March and site 8 in May (Fig. 5a). The highest value of nitrate recorded was 0.74 mg/L in September at site 3. The lowest nitrate concentration was recorded at site 1 in February (0.07 mg/L) (Fig. 5b). Ortho-phosphate concentration was highest in October at site 1 (0.13 mg/L). The lowest reading recorded was 0.01 mg/L in January at site 8 (Fig. 5c). The highest concentration of total suspended solids was 26.0 mg/L in February and June at site 1. The lowest concentration was 18.4 mg/L at site 4 in September (Fig. 5d).

### Canonical Correspondence Analysis of Cyanobacteria Communities and Environmental Variables

CCA was carried out to determine the relationships between cyanobacteria diversity and the environmental descriptors recorded. Two separate CCA are presented here. The first examines the relationship between the purely physical paraments, including type of substratum (bedrock, boulder, cobble, pebble, sand/silt), type of water flow (run, riffle, pool) and degree of shading (shaded, partly shaded, unshaded), with morphospecies diversity. The second examines the relationship of physicochemical parameters, including nitrate, ortho-phosphate, ammonia, total suspended solids, dissolved oxygen, temperature and pH with morphospecies diversity.

**Figure 6:**
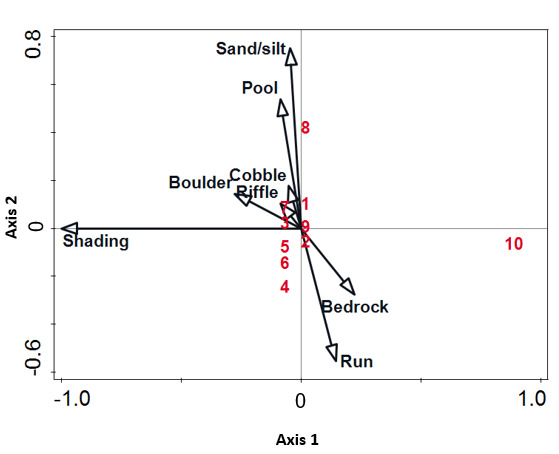
Canonical correspondence analysis biplot depicting the relationship between different physical environmental variables (type of substratum, shading and water flow) and the presence of cyanobacteria over the study period. (1) *Chroococcus* cf. *cohaerens*; (2) *Homoeoptyche repens*; (3) *Scytonema hofmanni*; (4) *S*. *stuposum*; (5) *Aphanocapsa* cf. *musicola*; (6) *Microcystis* cf. *firma*; (7) *Nematoplaca incrustans*; (8) *Phormidium simplicissimum*; (9) *Gloeocapsopsis* cf. *crepidinum*; (10) *Leptolyngbya valderiana.*

The CCA plot (Fig. 6) showed that the physical environmental variables explained 85.6% of variation in cyanobacteria diversity (*P* = 0.002; *pseudo-F* = 254). The first axis indicated that a large proportion of cyanobacteria showed preference for partly-shaded habitats. *Leptolyngbya valderina* was an exception to this generalisation and was located in the positive part of the first axis, showing a high affinity to light consistent with it being identified in an unshaded habitat. The type of substrata and water flow also affected the distribution of cyanobacteria. *Phormidium simplicissimum* showed a positive affinity for sand/silt and pool along Axis 2. This epipelic morphospecies was encountered on boulders and small roots coated with silt in a pool area. *Scytonema hofmanni* and *Nematoplaca incrustans* were correlated with boulders and cobbles, together with riffle, and others were correlated with bedrock and run.

**Figure 7:**
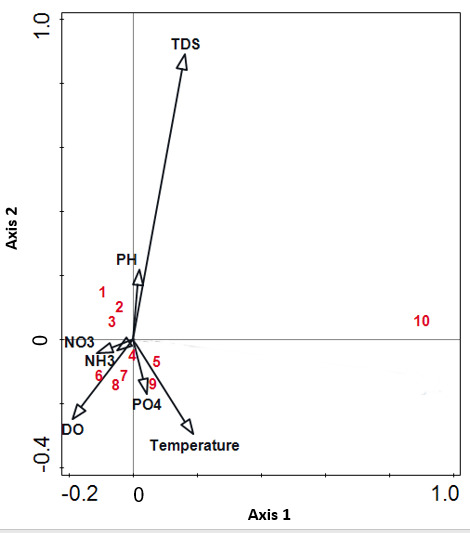
Canonical correspondence analysis biplot depicting the relationship between different physicochemical environmental variables (total suspended solids, dissolved oxygen, pH, temperature, light intensity, nitrate, ortho-phosphate and ammonia) and the presence of cyanobacteria morphospecies over the study period. (1) *Chroococcus* cf. *cohaerens*; (2) *Homoeoptyche repens*; (3) *Scytonema hofmanni*; (4) *S*. *stuposum*; (5) *Aphanocapsa* cf. *musicola*; (6) *Microcystis* cf. *firma*; (7) *Nematoplaca incrustans*; (8) *Phormidium simplicissimum*; (9) *Gloeocapsopsis* cf. *crepidinum*; (10) *Leptolyngbya valderiana*.

The second CCA (Fig. 7) showed that the physicochemical environmental variables explained a lower proportion, 14.6%, of variation in cyanobacteria diversity (*P* = 0.002; *pseudo-F* = 6.4). On Axis 1, *Scytonema stuposum*, *Microcystis* cf. *firma*, *Nematoplaca incrustans* and *Phormidium simplicissimum* showed positive correlation with nitrate and ammonia. On Axis 2, *Chroococcus* cf. *cohaerens*, *Homoeoptyche repens* and *Scytonema hofmanni* showed a positive correlation with pH, and *Aphanocapsa* cf. *musicola* and *Gloeocapsopsis* cf. *crepidinum* showed a positive correlation with phosphate concentration.

## DISCUSSION

A total of 24 morphospecies (11 unicellular, six filamentous and seven heterocytous) from four orders, nine families and 15 genera were recorded for the first time from Tukun River, Penang National Park, Malaysia, considerably increasing the known diversity of this area and of Malaysia. Unicellular taxa dominated (46%) the morphospecies encountered in the study stream, with lower contributions from heterocytous forms (29%) and simple filamentous (25%) cyanobacteria. A majority (70%) of these are new records for Malaysia. The high number of new records from this study further emphasises the current lack of knowledge in periphytic cyanobacteria in flowing waters in Malaysia.

The dominance of unicellular taxa in the study stream is underlain by their macroscopic growth forms. Bedrock, boulders and pebbles are more prevalent in riffles and runs throughout the study site. Sand and silt were more restricted to areas only with very slow runs. These unicellular taxa form the epilithic communities which are typically crust formers and possess the ability to adhere strongly on stable substrata and avoid sloughing caused by water movement. With the abundance of the suitable substrate coupled with the fast water current along Tukun River, the development of the epilithic community is more diverse than the epipelic ones which composed of the mat forming taxa.

Crust components recorded in field samples in this study included seven morphopsecies, of which *Homoeoptyche repens* and *Nematoplaca insrustans* are rare and poorly known species worldwide. Only two morphospecies of *Homoeoptyche* and one of *Nematoplaca* have been described (Komárek 2013). Morphological identifications of specimens obtained in the current study are consistent with the descriptions of the previously recorded morphospecies, originating from tropical regions in Guyana, South America, and Central Sumatra, Indonesia (Komárek 2013). This suggests a possibility of these morphospecies being cosmopolitan in the tropics, while also emphasising the lack of studies on crust-forming cyanobacteria in freshwater ecosystems generally.

Of the diversity obtained, a total of 14 morphospecies (identified using ‘cf.’) did not conform precisely to existing morphospecies descriptions. Morphological identification can be difficult because of the high phenotypic variability among similar cyanobacterial morphospecies observed under different environmental conditions (Komárek 2006). The lack of identification keys for the specific region again indicates the under-represented data from tropical and especially Asian regions (Komárek & Anagnostidis 1999, 2002; Komárek 2013). On this basis, the application of molecular methods in identification and phylogeny of cyanobacteria has been proposed as a tool to reveal the true diversity of the group (Komárek 2013). However, Komárek (2013) did not exclude morphological identification as the basis of evaluating cyanobacteria diversity, rather proposing a combination of approaches including both morphological and molecular evaluation for improved species identification. In the current study, morphological evaluation was employed to identify the diversity of cyanobacteria and thereby provide baseline information for a region with no available records. Further detailed investigations of ultrastructure along with molecular characterisation of these strains will be required to reveal the true diversity of the cyanobacterial microflora in Tukun River and in Malaysian ecosystems generally.

Members of the genus *Scytonema* were the most widespread throughout the study stream. Two morphospecies, *S. hofmanni* and *S. stuposum*, displayed different macroscopic growth forms, crust and mat, respectively. *S. hofmanni* co-occurred with other cyanobacteria including *Aphanocapsa* cf. *musicola, Microcystis* cf. *firma*, *Chroococcus* cf. *cohaerens*, *Nematoplaca inscrustans* and *Homoeoptyche repens* in the crust. In constrast, *S. stuposum* occurred as monospecific woolly mats. Dominance of single morphospecies in mats was also observed in mats of *Leptolyngbya valderiana* and *Phormidium simplicissimum*. Although many different morphospecies of cyanobacteria have the ability to co-occur in microbial mats, there is often domination by one species (Stal 1995) although the factors responsible for this are not well understood.

The genus *Scytonema* is characterised by the ability to fix nitrogen, containing heterocytes, and is generally considered to be cosmopolitan due to this ability (Komarek 2013). The distribution of heterocytous forms might be an indicator of lower environmental nitrogen levels (Saha *et al.* 2007). In the current study, the two *Scytonema* morphospecies recorded showed distinct distribution patterns. *S. hofmanni* was dominant in the upper to middle stream sites while *S. stuposum* was dominant in the middle and lower sites. The low nitrate concentrations (0.07–0.74 mg/L) recorded throughout the study stream facilitate the occurrence of both morphospecies throughout the Tukun River with the exception of site 9. The distinct separation in the distribution between the two *Scytonema* species observed here is attributed to their macroscopic growth forms and substrate availability. The distribution of the crust forming *S. hofmanni* was confined to the upper and middle reaches due to better cell adhesion on the stable substrata available. As for the easily displaced mat-forming *S. stuposum*, the preference for the soft sediments present in slow flow areas in the middle and lower reaches would help prevent displacement due to scouring. The differences observed here between the two morphospecies appears to be underlain by their ability to adapt to the particular microhabitat. Similar patterns in preferences for different substrata by different periphyton species have been describe previously (Downes *et al.* 2000; Murdock & Dodds 2007; Sekar *et al.* 2004) but no such records are available for cyanobacteria in Malaysian flowing water ecosystems.

Periphytic cyanobacteria in Tukun River had greater diversity in partly-shaded areas as compared to exposed areas. Six morphospecies were recorded in both shaded and partly-shaded sites (*Aphanocapsa* cf. *musicola, Microcystis* cf. *firma, Chroococcus* cf. *cohaerens, Scytonema hofmanni, Nematoplaca inscrustans* and *Homoeoptyche repens*). A further three morphospecies, *Gloeocapsopsis* cf. *crepidum, Phormidium simplicissimum* and *S. stuposum*, were encountered in partly-shaded areas alone. *Aphanocapsa* cf. *musicola* and *Chroococcus* cf. *cohaerens* represent genera which have been recognised for their adaptation to low light levels (Komárek & Anagnostidis 1999).

Even though cyanobacterial occurrence is generally greater in unshaded river environments (Branco & Necchi 1996), reports of their occurrence exist in lotic environments with varying degrees of shading, ranging from open to heavily shaded streams (Borges & Necchi Jr 2006). In the present study, the only site that was completely exposed was site 9. Although light penetration was greater here, the occurrence of morphospecies was low due to the constant change in salinity. Low light adaptation in cyanobacteria has been reported in many freshwater periphytic communities (Pentecost & Whitton 2012). For instance, colonial unicellular genera in heavily shaded streams are capable of producing abundant mucilage that allows colonies to remain hydrated and metabolically active for longer periods, assisting adaption to low light regimes (Pentecost & Whitton 2012). Many cyanobacteria species also possess phycobilin pigments in their photosynthetic pigment complex that enable them to thrive in low light levels (Krupek *et al.* 2007).

In contrast, salinity tolerance in freshwater cyanobacteria is not common (Otsuka *et al.* 1999; Tonk *et al.* 2007). The only morphospecies reported to be able to withstand salinity fluctuation is the toxin producing *Microcystis aeruginosa*, where blooms of this species have been reported to invade brackish water (Tanabe *et al.* 2018). *Leptolyngbya valderiana* was the only morphospecies encountered in the tidally inundated Site 9. *L. valderiana* has previously been recorded from brackish and marine waters (Komárek 2013), although it has also been recognised that this species’ taxonomy requires revision (Komárek & Anagnostidis 2002). Fluctuation in salinity coupled with the limited substrate availability may therefore be the controlling factors for occurrence and growth at this location

This study provides a first detailed account of cyanobacteria diversity and ecology in a Malaysian catchment. It provides a basis for long-term monitoring at this site of the effects of human activities in the catchment. Some cyanobacteria receive attention as a problematic symptom of eutrophic conditions through the occurrence of blooms. However, cyanobacterial occurrence is not always related to an ecological problem, and some species are characteristic of clean waters and can be used as bioindicators for monitoring the quality of running waters (Mateo *et al.* 2015). The widespread occurrence of heterocytous morphospecies and of rare and poorly known species suggests that parts of Tukun River remain relatively pristine.

## CONCLUSIONS

Twenty-four cyanobacterial morphospecies were identified for the first time in Tukun River, Penang Island, Malaysia. Seventeen are new records for periphytic cyanobacteria in Malaysian stream ecosystems. Eighteen of these could potentially be new species or varieties of existing species. Future molecular evaluation together with morphological assessment are required to reveal their full identity. The current lack of comprehensive cyanobacterial inventories and understanding of their distribution patterns in Malaysian flowing water ecosystems hinders the ability to utilise them as excellent bioindicators of ecosystem health.

## Figures and Tables

**Table 1:** List of morphospecies identified. Different macroscopic growth forms and the presence of the cyanobacteria strains in field samples and both clonal and mixed cultures are indicated. (+) = Present (−) = Absent.

Morphospecies	Field	Culture		Macroscopic form	
	
		Clonal	Mixed	Crust	Mat
SYNECHOCOCCALES					
*Aphanocapsa* cf. *musicola* (Meneghini) Wille	+	−	−	+	−
*Leptolyngbya* cf. *boryana* Anagnostidis *et* Komárek	−	−	+	−	−
*L.* cf. *foveolara* (Gomont) Anagnostidis *et* Komárek	−	+	−	−	+
*L. valderiana* (Gomont) Anagnostidis *et* Komárek	+	−	+	−	−
*Pseudanabaena* cf. *frigida* (F. E Fritsch) Anagnostidis	−	−	+	−	−
CHROOCOCCALES					
*Chroococcus* cf. *cohaerens* (Brebisson) N*ä*geli	+	−	−	+	−
*C.* cf. *dispersus* (Keissler) Lemmermann	−	+	−	−	−
*C.* cf. *membraninus* (Meneghini) N*ä*geli	−	−	+	−	−
*C.* cf. *minor* (Kützing) N*ä*geli	−	+	−	−	−
*C.* cf. *minutus* (Kützing) N*ä*geli	−	−	+	−	−
*C.* cf. *varius* A. Braun	−	−	+	−	−
*Chroococcus* sp.	−	+	−	−	−
*Gloeocapsopsis* cf. *crepidinum* (Thuret) Geitler ex Komárek	+	−	−	+	−
*Gloeocapsa* cf. *rupicola* Kützing	−	+	−	−	
*Microcystis* cf. *firma* (Kützing) Schmidle	+	−	−	+	−
OSCILLATORIALES					
*Geitlerinema* cf. *tenuius* (Stockmayer) Anagnostidis	−	+	−	−	−
*Phormidium simplicissimum* (Gomont) Anagnostidis *et* Komárek	+	−	−	−	+
NOSTOCALES					
*Dolichospermum* sp.	−	+	+	−	−
*Fischerella* sp.	−	−	+	−	−
*Homoeoptyche repens* Skuja	+	−	−	+	−
*Nematoplaca inscrustans* Geitler	+	−	−	+	−
*Scytonema hofmanii* C. Agardh ex Bornet *et* Flahault	+	−	−	+	−
*S. stuposum* (Kützing) Bornet ex Bornet *et* Flahault	+	−	−	−	+
*Westiellopsis* sp.	−	−	+	−	−

**Table 2:** Cyanobacteria morphospecies identified from each sampling location.

Morphospecies	Sampling location								

	Upper stream			Middle stream			Lower stream		
		
	1	2	3	4	5	6	7	8	9
CHROOCOCALES									
*Aphanocapsa* cf. *musicola*	−	+	+	+	−	−	−	−	−
*Chroococcus* cf. *cohaerens*	+	+	+	−	−	−	−	−	−
*Gloeocapsopsis* cf. *crepidinum*	−	−	−	+	−	−	−	−	−
*Microcystis* cf. *firma*	−	+	+	−	−	+	−	−	−
OSCILLATORIALES									
*Leptolyngbya valderiana*	−	−	−	−	−	−	−	−	+
*Phormidium simplicissimum*	−	−	−	−	+	−	−	−	−
NOSTOCALES									
*Scytonema hofmanni*	+	+	+	+	+	−	−	−	−
*S. stuposum*	−	−	−	+	−	+	+	+	−
STIGONEMATALES									
*Homoeoptyche repens*	+	+	+	+	−	−	−	−	
*Nematoplaca inscrustans*	−	+	+	−	−	−	−	−	−

(+) = Present (−) = Absent
